# Integrating public health policies in the epidemiological modeling of hepatitis C with LEHC tool: application in Austria

**DOI:** 10.1007/s00508-020-01774-y

**Published:** 2020-12-17

**Authors:** Henrique Lopes, Ricardo Baptista-Leite, Diogo Franco, Roxana Pirker, Michael Gschwantler

**Affiliations:** 1 Institute of Health Sciences, Public Health Unit, Catholic University of Portugal, Palma de Cima, 1649-023 Lisbon, Portugal; 2 Faculty of Health, Medicine and Life Sciences, Maastricht University, Maastricht, The Netherlands; 3 Department of Internal Medicine IV, Wilhelminenspital, Vienna, Austria; 4 Sigmund Freud University, Vienna, Austria

**Keywords:** Hepatitis C, Modeling, Public Policies, Public Health, Gamification

## Abstract

**Background:**

Eliminating hepatitis C requires addressing issues other than medicines or therapies. Public health policies focused on the hepatitis C virus (HCV) must be emphasized and worked to know the impacts on its epidemiologic dynamics. This research aims to provide a tool to evaluate and simulate alternatives by redefining policies meeting specific needs in each country towards the HCV elimination target by 2030.

**Methods:**

The development of a gamified model with 24 public health policies focused on HCV was conducted to evaluate the impact of measures in the disease epidemiologic dynamics. The Let’s End HepC (LEHC) project encompassed key populations (people who inject drugs [PWID], prisoners, blood products and remnant population) in Austria and other countries, presenting prospects for every year from 2019 to 2030. The LEHC epidemiological model comprised an integrated solution for HCV, with adaptive conjoint analysis (ACA) and Markov chains constituting its main processes.

**Results:**

Despite Austria’s efforts towards achieving the HCV elimination goal by 2030, the LEHC model forecast quantitative analysis predicts that it is still not enough to meet the target; however, prospects are very optimistic if public health policies are adapted to the country’s needs, being possible to achieve the goal as early as 2026.

**Conclusion:**

In Austria, the LEHC tool allowed to forecast the HCV elimination year anticipation to 2026, instead of being achieved after 2030. This target will only be valid if adequate management of the 24 public health policies focused on this pathology is further implemented.

## Introduction

Hepatitis C is a silent disease that afflicts people all over the globe. This virus affects people according to unknown timetables, which imposes an important role on health services in terms of human resources, technology and capital. Its evolution is well-documented in the natural history of the disease [1].

Globally, the hepatitis C virus (HCV) is the seventh leading cause of hepatic mortality [2], often related to hepatocellular carcinoma. Transmission networks of the disease are often associated with significant costs, either being liver transplants or indirect costs.

One of the HCV characteristics is its concentration of higher prevalence rates among given population segments: people who inject drugs (PWID) [3–5], people who have been incarcerated [6–8], individuals who have received blood products [9, 10].

To address the public health issue of HCV, the WHO has defined an elimination goal for the disease by 2030, aiming at reducing disease incidence by 90% and mortality by 65% [11]. This target has set the discussion about targeting population groups that are more prone to be infected, with the concept being characterized as micro-elimination. There are currently over ten groups that meet this criterion [12]: individuals with Aboriginal and indigenous background, people born in specific periods, children whose mother was infected with HCV during pregnancy, hemodialysis patients, individuals coinfected with HCV/HIV, PWID, migrants from high HCV prevalence countries, people who have received blood products, prisoners, transplant recipients, war veterans [13], baby boomers [14], among others.

Projections for the HCV prevalence have been considered to be between 0.5% and 3.5% in Europe [15, 16] and 7–8% [17] in other continents. The presented values have been subject to updates in epidemiological revisions [18], with a decrease in HCV prevalence rates being verified to be 0.2–1.5% in Europe [19]. In Austria, it has been estimated that the prevalence of HCV infections was 0.46%, and the prevalence of viremic prevalence around 0.34% in 2008 [20]. For this reason, including Austria in the first modeling phase of the LEHC project was seen as essential.

The LEHC tool aims to evaluate and simulate diverse adaptations of public health policies according to the modeled epidemiological characteristics of the disease and the needs of a country to achieve the HCV elimination goal by 2030. By modeling the impact of the dynamics between both referred elements, it is possible to generate a landscape on HCV epidemiology for every year until 2030. Therefore, the model is also aligned with the sustainable development goals timetable. The study of the 24 health policies impact and their different levels of implementation resulted in different estimates on HCV epidemiology for different population groups: PWID, blood products, vertical transmission^1^, remnant population. The natural history of HCV is reflected in the indicators that result from the epidemiological dynamics on model projections.

This project is being promoted by the Public Health Unit of the Health Sciences Institute of the Catholic University of Portugal. Along with Austria, other countries were included in the project’s initial modeling phase (Romania, Bulgaria, Portugal, Spain) while others are also being currently worked.

## Methods

The LEHC model has the potential to forecast and gamify^2^ different applications of public health policies while calculating its impacts on HCV epidemiology. It is possible to assess the modeling results for six population groups: remnant population, prisoners, PWID, blood products, vertical transmission and total population. The remnant population was calculated according to the formula: remnant population = total population − (blood products population + prisoners and ex-prisoners + PWID and ex-PWID). Due to its low number, the vertical transmission population was included in the remnant population.

The LEHC epidemiologic model integrated a total of 1100 active Markov chains^3^ branches among the identified population groups, which resulted in data that characterize the HCV epidemiological evolution. Health states and respective transition probabilities are also included in the branches system, being defined according to the natural history of HCV as identified by Salomon et al. [1] in 2003. The Markov chains component is applied for the period between 1950 until the present time, featuring data that correspond to indicators, such as incidence/prevalence rates as well as diagnosis, retain in care, treatment and sustained virologic responses (SVR) rates. The availability of data regarding epidemiological indicators for HCV is linked with research in the mid-1990s, driven by the ground-breaking hepatitis C virus discovery in 1989. As a result, when no data were available or the existing information did not meet the full criteria for the statistical series, proxies were produced to atone the modeling components. Proxies were contemplated as assumptions, being assessed and validated by the country’s National Advisory Board (NAB) and were subject to the mathematical evaluation by comparing the model results with empirical data.

It is assumed that the statistical data used for the LEHC model are correct, considering that they are either collected from peer-reviewed journals or have their source in official data of countries or regions. In the case where it was not possible to retrieve data from these sources, proxies had to be produced, with the assumption that the leading experts in each country of the LEHC project are able to provide an accurate overview of the epidemiological reality.

However, it is known that even data that were subject to peer review have often existed solely in micro-populations while being related to the methodology used in data collection. It must be noted that even official data from countries has some frailties, such as the difference of the total number of inhabitants by over one million individuals, the source or country on the origin of the data, the opinion of the most acknowledgeable experts, which is always a personal view based on vast experience.

Around 100 key opinion leaders (KOL) from each NAB have also participated in surveys based on an adaptive conjoint analysis (ACA) approach, taking a global average of 2h for completion. The resulting data were used to develop the modeling component related to the 24 public health policies (additional details in htttp://www.letsendhepc.com/)^4^ in each country. These policies have a distinctive scale of implementation being contemplated on the surveys, in which every KOL attributes a different weight to different policy levels. This process allows the differentiation of forecast results by having distinctive impacts on the Markov chains component, according to a cure cascade^5^ perspective. Modeling in Austria was supported by the participation of seven KOLs in completing the ACA surveys.

## Model construction and data support

### Total population, remnant population and vertical transmission population

Austria’s demography for the total population, remnant population and vertical transmission was constructed by accessing data from the Statistics Austria website [21] concerning the country’s total population, live births and residents with foreign citizenship [22]. For each year, the total population should be the sum of last year’s total population plus the number of individuals who enter the population minus the number of individuals who leave the population.

For the background mortality series, the annual probabilities of death were also based on data from the Statistics Austria website [23].

New chronic HCV infections and HCV prevalence rates were extrapolated and adapted to a national perspective of Austria [20], being aligned with two other scientific reports [24, 25].

The vertical transmission comprised HCV incidence values that represented the total cases of HCV positive live births, being included in the remnant population. Based on the availability of data it was considered that there is a total of 19 or 20 cases in Europe per year. The values were adapted and extrapolated to fit Austria’s reality [26].

In Austria, hepatitis C-related diagnostic techniques were assumed to have begun implementation in 1997 [20]; therefore, a rate of 0% was applied to previous years. For the following years, diagnosis rates were presumed to be equal for both genders and for all the age groups, according to the Metavir score system, 5% in fibrosis stages F0–F3, 7% in F4, 95% in decompensated cirrhosis and 70% in hepatocellular carcinoma stage.

No data were found regarding the annual probability of a diagnosed patient being retained in care. Therefore, it was decided that the Portuguese data [27] for this indicator should be used to effectively represent the Austrian population.

In Austria, HCV treatments (Tx1) [20] were assumed to have started in 1997 in relevant numbers. Recent direct-acting antiviral (DAA) treatments (Tx2) [20] were assumed to have started in 2014. Data regarding SVR rates for patients who followed each treatment were also extrapolated from the study by Schaefer et al. [20].

### PWID population

The PWID demography was based on two reports [28, 29], with values being estimated by regression analysis. For the period between 2019 and 2030, values were aligned with the trend from previous years. In order to determine the number of individuals who start/stop injecting drugs each year, data from the “Suchthilfe Wien” (personal communication, unpublished data, https://suchthilfe.wien) were used along with the aforementioned reports.

Data regarding the annual probability of death were based on the working group drug-related fatalities (2017) report (“Arbeitsgruppe drogenbezogene Todesfälle (Sterbejahr 2017)”, [30]) for the period between 2003 and 2017. Considering the identified trend, data for the missing years were accordingly aligned.

Values for HCV prevalence and HCV incidence were based on scientific reports [28, 29]. A report from the EMCDDA [31] estimated a prevalence of 30,000–33,000 high-risk opioid users in Austria. The percentage of chronic HCV infections ranged from 13–83% in 2017.

Diagnosis rates were considered to be 0% before 1997. For the following years, diagnosis rates were produced according to data from the “Suchthilfe Wien” (personal communication, unpublished data, https://suchthilfe.wien). For the period between 1997 and 2018, diagnose rates for both genders, and all age groups were assumed to be 1% for F0–F4 stages and 80% for decompensated cirrhosis and HCC stages.

The annual probability of an individual being retained in care if the Hepatitis C was already diagnosed was determined in statistical series under qualitative advice of the NAB in Austria.

The annual probability of an infected individual to be treated for the first time against HCV if already retained in care was based on the “Suchthilfe Wien” (personal communication, unpublished data, https://suchthilfe.wien). Until 1996 it was assumed that therapies were not yet available.

As for the annual probability of an infected individual being retreated against HCV if already retained in care, data were based on the “Suchthilfe Wien”. It was assumed that DAA therapies were not yet available before 2014. For the period between 2014 and 2018, a rate of 20% was assumed for both genders and age groups in each stage of liver disease.

The success rates of the patient’s first treatment and retreatment with DAA therapies were determined under the qualitative advice of the Austrian National Advisory Board.

### Prisoners population

Data retrieved from the Ministry of Justice [32] were used to produce the demography’s statistical series for the prison population. For the period between 1950 and 1982, data were considered to be constant, while between 2019 and 2030, the trend observed in previous years was adapted and used as a reference.

For the period between 1950 and 2030, data concerning this population’s background mortality was based on the values used in the remnant population regarding the same indicator. It was assumed that the probability of death while in prison was three times higher as compared to the general population.

It was assumed that 1% of all prisoners were newly infected with HCV each year, for the period between 1950 and 2018. It was assumed that 15% of all prisoners were chronically infected with HCV.

Annual probabilities of an infected individual being diagnosed were produced according to the qualitative advice of the National Advisory Board in Austria.

Until 1996 it was assumed that retain in care (RIC) was not feasible. Data from the Ministry of Justice (oral communication, unpublished data) were used for the period between 1997 and 2018. The RIC rates for both genders and all age groups were assumed to be 20% for F0–F4 stages, 70% for decompensated cirrhosis stage and 60% for the hepatocellular carcinoma stage.

Annual probabilities of individuals being treated for the first time and/or retreated for HCV, along with the respective sustained virologic response rates were produced under the advice of the National Advisory Board.

### Blood products population

This population group encompasses the total number of individuals that have ever received a blood transfusion.

Demography values were based on data from Red Cross Austria (https://www.roteskreuz.at/). A total of 350,000 blood products were used in 2017. It was estimated that each patient who receives blood products uses an average of three blood preserves, according to clinical estimates. Values were assumed to be increasing in the period between 1950 and 2010. Due to the implementation of new programs and therapies, it was assumed that, for the remaining years, a decreasing rate should be applied to the identified trend.

The background mortality rates were considered to be similar to those in the remnant population. Considering that blood screening for transfusions was introduced in Austria in 1990, data were accordingly adjusted to represent its impact on mortality.

It was assumed that half of the HCV infected population in Austria was due to blood products during the period between 1950 and 1983. The probability of infection by HCV was based on an extrapolation from the general population values. For the period between 1983 and 1990, a decrease was applied in order to reach a probability of infection by HCV value of 0.001. This value is expected to have remained constant for the remaining years.

HCV prevalence values are assumed to be of constant increment for the period between 1950 and 2010, decreasing in the following years in order to be aligned with a prevalence of around 3% of the blood products population in 2018.

Diagnosis rates were assumed to be equal to the ones used for the remnant population. In the period between 1997 and 2018, values for both genders and all age groups were assumed to be 5% from F0–F3 stages, 7% in the F4 stage, 95% in decompensated cirrhosis stage and 70% for hepatocellular carcinoma stage.

Retain in care values were also assumed to be the same as in the remnant population. A rate of 80% was considered for all liver disease stages in both genders and all age groups.

The annual probability of an individual being treated for the first time and/or retreated against HCV if retained in care was assumed to be the same as for the remnant population. The same process was conducted with SVR of first treatment and retreatment.

## Results

### Epidemiological indicators

In Austria, the model results for the current year (i.e., 2019) appoint a total population of 8,679,051 individuals, with 29,348 being infected with HCV and an occurrence of 607 new cases is to be registered. Forecasts show that in 2019 around 16,626 individuals will be diagnosed, with 12,402 being linked to care, from which 5836 will be under treatment and 1064 cured cases are expected to happen. As for the different liver disease stages, model results for this year show that there will be 4934 cases of compensated cirrhosis, 337 decompensated cirrhosis and 206 cases of hepatocellular carcinoma. In addition, 11 liver transplantation cases are expected, as well as 202 liver-related deaths.

**Fig. 1:**
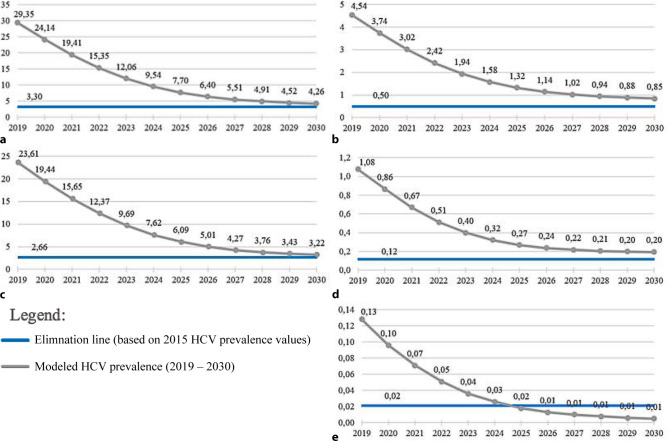
Modeled HCV prevalence for the studied populations in Austria. **a** Total population summary. **b** Remnant population and vertical transmission. **c** PWID + ex-PWID. **d** Prisoners + ex-prisoners. **e** Blood products. The presented values (thousands) consider the forecast of current policies in Austria remaining unchanged for the period between 2019 and 2030. Comparison with the elimination target for each population (total population, remnant population, vertical transmission, blood products, prisoners, PWID)

The LEHC model forecasts that HCV elimination will not be achieved by 2030 (see Fig. 1). In that year, the total population is expected to comprise 8,532,313 individuals, from which 4261 are infected with HCV and 607 are estimated to be new cases. Considering the pool of HCV infected individuals, around 2621 are predicted to be diagnosed with 1883 being linked to care. As a result, in 2030, 784 individuals are expected to be on treatment. In the same year, 810 individuals should be cured. Concerning the different liver disease stages, the model forecasts 4351 cases of compensated, 104 cases of decompensated cirrhosis and 82 cases of hepatocellular carcinoma. Due to the burden of disease, 3 liver transplantations are expected to be conducted, and it is estimated that there will be 82 cases of liver-related deaths.

### Modeled HCV prevalence by population

The LEHC model estimates that HCV prevalence values will steeply decrease until 2025 and slightly decrease until 2030. Assuming the elimination line as 10% of the modeled HCV prevalence value in 2015, the WHO target will not be achieved by 2030 (see Fig. 1). Despite modeled HCV prevalence values for 2030 being very close to reaching the elimination line, the worst-case scenario was considered in the face of an uncertainty zone as a conservative reading of results. This reading was based on not incurring in risks of indicating results that could influence decision-makers, legislators and national health authorities to over-alleviate public health policies focused on Hepatitis C. The two points of uncertainty that can improve this reading will be to define more accurately the real number of HCV infected individuals in Austria in 2015 (reference value for the WHO elimination target by 2030), which in the case of Austria is particularly fragile compared to the remaining five countries participating in the LEHC project. Also, forecasting for a 12 years period implies a high uncertainty margin, reason why the LEHC project seeks to revisit national data, whenever there is new concrete data that allows to recalibrate the model.

Modeled HCV prevalence values for the remnant population and vertical transmission are expected to slightly decrease over the years. The elimination target for this population will not be achieved by 2030 (see Fig. 1).

As for the blood products population, HCV prevalence is also expected to decrease over the years. According to the LEHC model, the WHO target for this population will be achieved in 2025 (see Fig. 1).

HCV prevalence for the prisoner population is expected to steeply decrease until 2025 and slightly decrease over the remaining years. HCV elimination will not be achieved by 2030 in this population (see Fig. 1).

It is expected that HCV prevalence values for the PWID population will gradually decrease until 2027 and slightly decrease over the remaining years. HCV elimination will not be achieved by 2030 for this population (see Fig. 1).

### Policies

Forecast results from the first version of the LEHC model indicate that if there are no changes in the 24 public health policies in Austria, HCV will not be eliminated in the country by 2030. On the other hand, it is still possible to improve 9 of the 24 public health policies (see Table 1 and Appendix, Table 2).

**Table 1:** Public health policies differential gains on the reduction of HCV infection cases in total population, in 2030, if fully implemented

Policy impact on total population in 2030	
Public health policies	Reduction in HCV infection cases
National strategy that includes the HCV (evaluation of available resources)	913
National strategy that includes the HCV (clinical evaluation)	761
National policy to address the prevention of HCV infection	470
Legal framework particularly in terms of discrimination of patients with HCV	385
Screening for HCV in tissue and organ donations	234
Routine screening of HCV in pregnant women	108
Screening in the general population	98
Test sites/screening HCV outside the hospital environment	75
Risk reduction services in prisons	10

The reduction in HCV infected cases, in each policy, are not cumulative

The represented value illustrates the impact that each policy, if fully implemented, can have in the estimates for the year 2030

The policies in this table are organized by impact level on the reduction of HCV infection cases, in descending order from top to bottom

It is possible to achieve the same result by 2028 if a national strategy that includes the evaluation of available resources and clinical evaluation is implemented.

In addition to the changes in the aforementioned policies, it is possible to achieve an earlier HCV elimination by fully implementing sets of three other policies that focus on the dimensions of awareness and prevention. For example, this result can be achieved by extending the legal framework in terms of discrimination of patients with HCV. It could also include access to services, social and public facilities, instead of just having a labor framework; by developing and fully adopting the national policy to address the prevention of HCV infection and finally, by implementing a policy of impeditive donation in screening for HCV in tissue and organ donations, instead of not hindering donation.

By fully implementing all the 24 policies in Austria, the model results present the possibility of achieving HCV elimination by 2026. For that year, according to this optimal prospect, there will be a total population of 8,596,428 individuals. HCV incidence values are expected to be 343 cases and HCV prevalence around 2632 HCV infected individuals. The model also forecasts 1927 diagnosed individuals, with 1587 being linked to care and 756 enrolled in treatment. In the same year, estimates indicate 982 cured cases. In relation to liver disease stages, the model predicts 4685 compensated cirrhosis, 142 decompensated cirrhosis and 99 individuals with hepatocellular carcinoma. It is expected that 4 liver transplants are conducted, and an occurrence of 108 liver-related deaths is to be registered.

Considering the full implementation of the 24 policies in Austria, it is possible to achieve additional benefits for the years after 2026. A decrease in the total population is appointed by the LEHC model for 2030, presenting 8,532,441 individuals in Austria. HCV incidence values remain the same, with 343 new infection cases. HCV prevalence is forecast to significantly decrease, with 1843 HCV infected individuals. The model appoints 1153 diagnosed patients, with 885 being linked to care and 384 on treatment. In that year, results appoint for 386 cured individuals. In relation to liver disease stages, it is forecast that there will be close to 4109 cases of compensated cirrhosis, 82 cases of decompensated cirrhosis and 71 cases of hepatocellular carcinoma. Finally, the model results show the possibility of 2 liver transplantations being conducted as well as the occurrence of 67 liver-related deaths.

## Discussion

Modeling hepatitis C in Austria was based on gathered data from multiple sources, such as national entities (Statistics Austria, Suchthilfe Wien, Ministry of Justice, Red Cross Austria, etc) [21–23, 32]. and published studies [20, 24, 25, 31]. When no data were found to complete statistical series elements such as specific years, stratification by age or by gender, proxies were produced as detailed in the methodology. Estimates were based on both national and international studies regarding the populations covered by the LEHC project: Remnant population and vertical transmission [20, 24–26], people who inject drugs [28, 29, 31], prisoner populations [32], blood products population.

Empirical evidence resulting from the LEHC project corroborates the view that the path for eliminating hepatitis C until 2030 requires a higher focus on finding and maintaining patients within the grasp of the healthcare system. Given the high prevalence and incidence rates among population groups, such as PWID and prisoners, it is important that measures focus on the micro-elimination concept.

It is also important to consider the risk of overlapping populations, especially between PWID and prisoner populations, and to a much lesser extent between PWID and blood products. It was not possible to find published epidemiological data that could separate these populations in the remaining LEHC countries. As a result, in the modeling process, a proxy was created whereby it is assumed that about 60% of the prison population would have been HCV-infected in the past by i.v. drug use, subtracting this number from the PWID population. A similar situation was applied to the relationship between blood products and PWID, seeking to correct double interpretation situations.

Considering the modeling results, there are several situations in which the number of patients with compensated cirrhosis is higher than the total number of HCV infected individuals. This situation is verified as people who are successfully treated for HCV will still have pre-existing conditions such as the cirrhotic state. Considering the increasing number of people undergoing treatment and thus cured, it is natural that even with new HCV infection cases, in a given moment there will be less HCV infected individuals than individuals with compensated cirrhosis.

In addition, diagnosis and linkage to care measures are paramount to meet the HCV elimination target, being aligned with the sustainable development goals. In Austria, it has been confirmed that 15 out of the 24 public health policies considered for the study are currently fully implemented.

Despite the fact that other countries are also included in the LEHC project, it was considered that introducing external readings of comparison between country policies and epidemiological indicators could be an inducement of bad practices for health authorities. In the LEHC project’s developed work in 12 countries, the dynamics of public health policies were verified to be very independent, even in common issues.

## Conclusion

The LEHC project accomplished the modeling of hepatitis C in terms of public health policy impact on the disease epidemiology for the remnant population and other population groups in Austria. The dynamics of this tool allowed the gamification of public health policies, which provided an insight into how different extents of specific measures have an impact on the HCV epidemiology.

This model has the ability to forecast the extent to which a specific public health policy might exert influence on a particular population group, in a pool of 24 public health policies focused on HCV considered in the LEHC project. This is essential in identifying patterns that have the potential to not only allow countries to address given policies to achieve the HCV elimination goal by segmenting the population but also to know the why, who, what, where and when. The tool allows rehearsing public health policy concepts before implementing them in the field, thus anticipating the outcomes.

In the light of the cure cascade system, modeling results appoint awareness and prevention as the dimensions in which public health policies might have the broadest impact in lessening HCV prevalence values in Austria. Therefore, given these areas of influence, there is a clear indication that achieving the HCV elimination target will require additional efforts besides medication development. Diagnosing HCV in individuals who are unaware of their condition and linking them to healthcare services will determine the success of achieving the HCV elimination goal.

According to the modeling results, the elimination target will not be met by 2030 in Austria. Nonetheless, in the case that all the 24 public health policies are fully implemented, it will not only be possible to meet the elimination target, but it might happen as soon as 2026. As a result, it is seen that policies are fundamental in providing insights into combating HCV in order to achieve its elimination.

## Research limitations

The quality of the modeling always depends on data quality used as input, on the quality of the analysis and on the knowledge about the public health policies’ impact in the field. In LEHC (or any other model), it is paramount to deal with the issue of quality as well with data availability.

Since there are no confidence intervals in modeling, the fitting test was performed by overlapping the results obtained by the LEHC modeling tool, on the known epidemiological curve or admitted into a given geographic region, in this case, a country. In the LEHC tool, only countries where this curve was at least 95% similar in the previous years were selected for publishing data; however, it must be noted that even national data are often limited in their reading due to being either very regional (in the case of Austria, most of the information comes from Tyrol), from samples sometimes incident on populations that do not represent the country (studies with prisoners or PWID), or epidemiological studies that do not cover the entire population. This dependence on adjusted fitting based on known epidemiological evidence is a limitation of the tool, but endogenous to the modeling process. No country which worked in the LEHC project showed strong robustness of epidemiological data.

Considering that the LEHC model was based on the best available data from Austria, it is also important to state that obtaining national data was challenging, which imposed the need of producing proxies from Austrian regions, to extrapolate and interpolate national data, and to create statistical series having proxies as a basis composed by other secondary data series. Therefore, the Austrian LEHC model is the best possible to achieve, considering the data available. A call for action on the research and publication of epidemiological data regarding hepatitis C in Austria is of the utmost importance in filling in data gaps. This project is fully available to analyze and integrate all data that the scientific community provides for this project.

In perspective, the dynamic vision for this project should contemplate an annual review for integrating new official data and other epidemiological data that might have been produced in the meantime. Similarly, the authors comprise the modeling results of LEHC for 31 December 2018, in which it is based on statistical data available up to the referred data and this being designated as “LEHC: An integrated solution for HCV—application in Austria”.

## Appendix

**Table 2:** Main Public Health Policies differential gains in reducing HCV infection cases, in 2030, if fully implemented, by population

	Main policy impacts in reducing HCV infection cases in 2030			
Public health policies	Remnant population	Blood products	Prisoners	PWID
National strategy that includes the HCV (clinical evaluation)	159	1	39	658
National strategy that includes the HCV (evaluation of available resources)	153	1	38	723
National clinical guidelines for the diagnosis and treatment of HCV	78	n.a.	n.a.	n.a.
Legal framework, particularly in terms of discrimination of HCV patients	74	0	18	367
National policy to address prevention of HCV infection	n.a.	0	23	447
Screening for HCV in tissue and organ donations	57	0	11	223
Risk reduction services available in prisons	0	0	10	0
HCV testing/screening sites outside of the hospital environment	11	0	4	72
General population screening	38	0	0	98
Routine screening of HCV in pregnant women	42	0	0	108

The reduction in HCV infected cases, in each policy, are not cumulative

The represented value illustrates the impact that each policy, if fully implemented, can have in the estimates for the year 2030

*n.a.* not applicable

## Abbreviations

ACA: Adaptive conjoint analysis
DAA: Direct acting antiviral
EMCDDA: European Monitoring Centre for Drugs and Drug Addiction
F0–F4: Metavir stages
HCV: Hepatitis C virus
HIV: Human immunodeficiency virus
ICS/UCP: Healthcare Sciences Institute/Catholic University of Portugal
KOL: Key opinion leaders
LEHC: Let’s End HepC
NAB: National Advisory Board
PWID: People who inject drugs
RIC: Retain in care
SVR: Sustained virologic response
Tx1: first treatment for chronic hepatitis C
Tx2: The probability of an infected individual being retreated against HCV if already retained in care and previously treated
WHO: World Health Organization

## Footnotes

1 HCV transmission from pregnant mother to child.

2 Simulator that in a technologically opaque way allows the user to create and test results over time for theoretical scenarios.

3 Mathematical tool that uses successive multiplications for describing a phenomenon with time existence, and simultaneously with topologic intersection over the time variable.

4 Additional information is available in the preprint article under submission “LEHC—Public Health Policies impact in HCV epidemiological modeling: a tool towards a micro-elimination path.” [33].

5 Strategy to approach pathologies used in public health, in general by all major health organizations that is based on successive and logical focal points of disease management: awareness, prevention, diagnostics, linkage to care, treatment, reintegration.
